# Microstructure and coupling mechanisms in MnBi–FeSiB nanocomposites obtained by spark plasma sintering

**DOI:** 10.1038/s41598-024-67353-7

**Published:** 2024-07-24

**Authors:** A. Alexandru-Dinu, C. Locovei, C. Bartha, M. A. Grigoroscuta, M. Burdusel, A. Kuncser, P. Palade, G. Schinteie, N. Iacob, W. Lu, D. Batalu, P. Badica, V. Kuncser

**Affiliations:** 1 National Institute of Materials Physics, Street Atomistilor 405A, 077125 Magurele, Romania; 2 Faculty of Physics, University of Bucharest, Street Atomistilor 405, 077125 Magurele, Romania; 3 Shanghai Key Laboratory of D&A for Metal-Functional Materials, School of Materials Science and Engineering, Tongji University, Shanghai, 201804 China; 4 Faculty of Materials Science and Engineering, National University of Science and Technology Politehnica Bucharest, Splaiul Independentei 313, 060042 Bucharest, Romania

**Keywords:** Rare-earth-free permanent magnets, MnBi–FeSiB nanocomposites, Coupling mechanisms, Spark plasma sintering, Magnetic properties and materials, Materials science

## Abstract

Fabrication and extensive characterization of hard-soft nanocomposites composed of hard magnetic low-temperature phase LTP-MnBi and amorphous Fe_70_Si_10_B_20_ soft magnetic phase for bulk magnets are reported. Samples with compositions Mn_55_Bi_45_ + x⋅(Fe_70_Si_10_B_20_) (x = 0, 3, 5, 10, 20 wt.%) were prepared by spark plasma sintering of powder mixtures. Characterization has been performed by X-ray diffraction, scanning and transmission electron microscopy, magnetometry and ^57^Fe Mӧssbauer spectroscopy. It was shown that samples contain crystallized and nanometric LTP-MnBi phases with various elemental compositions depending on the degree of Bi clustering. Complex correlations between starting compositions, processes during fabrication, and functional magnetic characteristics were observed. Unexpected special situations of the relation between microstructure and magnetic coupling mechanisms are discovered. Exchange spring effects of different strengths occur, being very sensitive to morpho-structural and compositional features, which in turn are controlled by processing conditions. An in-depth analysis of related microscopic characteristics is provided. Results of this work suggest that fabrication by powder metallurgy routes, such as spark plasma sintering of hard and soft magnetic powder mixtures, of MnBi-based composites with exchange spring phenomena have a high potential in designing and optimization of suitable materials with tunable magnetic properties towards rare-earth–free permanent magnet applications.

## Introduction

Rare earth (RE) based permanent magnets, especially Nd_2_Fe_14_B and Sm-Co compounds^1,2^, play an important role in different modern-day systems such as hard disk drives, sensors and actuators, electric vehicles, etc. However, due to the shortage and the high cost of rare earth elements^3^, efforts were focused on finding RE-free alternatives^4^.

Among the RE-free permanent magnets of high performance are the Mn-based permanent magnets^5^. In particular, MnBi, a ferromagnetic intermetallic compound with a hexagonal structure, is of interest mainly due to its high magneto-crystalline anisotropy of the low-temperature phase (LTP) and its positive temperature coefficient of coercivity^6,7^. To measure the efficiency of a permanent magnet, one has to determine its energy product (BH)_max_, which depends on the magnet’s coercivity and saturation/remanence. The theoretically estimated energy product for monocrystalline LTP MnBi as a thin film at 300 K is 20 MGOe^8^. By controlling the Mn/Bi ratio, an energy product (BH)_max_ of 16.3 MGOe has been experimentally achieved in a pure LTP MnBi *c*-axis oriented thin film^9^. However, in the bulk samples, because of the thermal decomposition of the ferromagnetic LTP MnBi over 628 K and segregation of Mn from the liquid phase below 719 K^10,11^, it is still challenging to fabricate MnBi magnets with both high content of LTP and large coercitivity^12,13^. For the bulk MnBi, the first principle calculations indicate a theoretical energy product (BH)_max_ of 17.7 MGOe at room temperature (RT)^14^. However, due to difficulties in preparing single-phase MnBi with controlled stoichiometry and without oxidation, lower values of about 8 MGOe have been reached experimentally^15–18^. Much attention should be paid to the optimization of the processing route and of the LTP-containing material. Arc-melting or hot compaction were employed to produce high-performance MnBi bulk magnets^13,15,19^. Also, spark plasma sintering (SPS) has shown promising results^20,21^. Some studies reported that structural, magnetic, and magneto-optical properties of MnBi bulks could be enhanced by using additions^12,22–24^.

This study proposes composites of MnBi–FeSiB and investigates the complex relationship between microstructural details and magnetic exchange coupling of the hard magnetic phase (MnBi) and the soft magnetic phase (FeSiB), to obtain a so-called “exchange-spring magnet”. Such systems are nanocomposites, which are built of hard and soft magnetic nano-phases with strong exchange interactions at the interface of the two phases^25–28^. They are promising for advanced permanent magnet applications since they have a larger (*BH*)_max_ compared to the weighted superposition of energy products of individual constituents and they allow new possibilities in designing and tuning of the functional characteristics of the permanent magnets.

It is also noteworthy that many articles report on using additives to generate the so-called doping effects by substitution or introduction into the interstitial sites of the MnBi crystal lattice of different elements rather than to produce hard-soft composites. For this purpose, in general, doped MnBi magnets are fabricated via different melting routes. On the other hand, for hard-soft MnBi-based composite magnets, solid-state fabrication methods might be cheaper and more appropriate, but the information in the literature is scarce. In the case of solid-state fabrication methods doping effects of the MnBi grains during fabrication of the hard-soft magnetic nanocomposites cannot be excluded either.

For the soft component, morpho-structural aspects are crucial in determining magnetic properties. Soft magnetic iron-boron-based amorphous alloys have been used in various electric devices involving high magnetic susceptibilities and low hysteretic losses^29^ because they have high saturation magnetization (*M*_s_) and low coercive field (*H*_c_). Yue et al.^30^ reported the compositional dependence of magnetic and thermal properties of melt-spun FeSiB amorphous alloys. They concluded that when increasing the content of Fe in the amorphous phase, the crystallization and Curie temperatures (*T*_c_) decrease drastically. Above room temperature, amorphous alloys with higher Fe content had reduced thermal stability. According to ref.^31^, for Fe–B–C–Si–P amorphous alloys, the value of *T*_c_ is predominantly determined by the Fe content, which also defines the strength of the exchange interactions in the alloy. Investigations from ref.^32^ have shown the effect of Cr addition on thermal stability and magnetic properties in Fe_79.5−x_Cr_x_Si_9.5_B_11_. Authors observed that by increasing Cr content, thus decreasing Fe content, the first crystallization temperature shifted towards higher temperatures, although the saturation magnetization decreased slightly. Remarkably, these types of alloys have a significant economic advantage due to their lower cost compared to the soft magnetic FINEMET type^33–35^ nanocrystalline alloys.

In the light of previous paragraphs, in this article, we report on fabrication by powder metallurgy and extensive characterization of hard-soft nanocomposite bulk magnets composed of hard magnetic LTP-MnBi and amorphous Fe_70_Si_10_B_20_ soft magnetic components. Namely, samples with compositions Mn_55_Bi_45_ + *x*⋅(Fe_70_Si_10_B_20_) (*x* = 0, 3, 5, 10, 20 wt.%) were prepared by SPS from powder mixtures. We found that the correlations between starting composition, processes during fabrication, and functional magnetic characteristics are complex and promote special situations. Microscopic mechanisms are discussed and highlighted e.g. versus microstructural details for a better understanding of the results and to provide a guiding line for the design and optimization of hard-soft composite magnets of this type that take advantage of exchange-spring phenomena.

## Experimental

Commercial high-purity manganese (99.99%) and bismuth (99.99%) (produced by Northeast Nonferrous Metals Market Co., Ltd., Shenyang, China) were used for obtaining a Mn_55_Bi_45_ ingot by induction melting in argon gas. The ingot was then cut into pieces of about 5 g. Further, the ingot was used to obtain Mn_55_Bi_45_ ribbons by single-roller melt spinning with a tangential speed of 40 m/s. The ribbons were annealed for 30 min at 573 K in vacuum. Then, ribbons were ground using a pestle and agate mortar and sieved through a # 300 mesh resulting the particle size down to less than 48 µm^36^.

Alloys with the nominal composition corresponding to Fe_70_Si_10_B_20_ were prepared by melt spinning of pressed Fe, Si, and B powder mixtures. The as-pressed sample was transferred into a quartz crucible with a capillary tube (diameter of 0.2 mm) at its bottom end. The capillary tube was placed at 2 mm from a 400 mm diameter Cu wheel, which was rotating with 2000 rot/min. After melting the sample in the crucible, the liquid was pushed through the capillary on the Cu wheel by applying an Ar over-pressure of 60 kPa. Following the interaction between the melt and the cooled wheel, ultra-fast solidification took place (cooling rate of 10^6^ K/s) and ribbons with about 1 mm width and about 30–50 μm thickness were obtained. The intermetallic ribbons were ground by using a high-energy ball milling system (SPEX 8000 M mixer/mill) with the ball to powder ratio of 10:1.

Samples with nominal compositions Mn_55_Bi_45_ + x⋅Fe_70_Si_10_B_20_ (x = 0, 3, 5, 10, 20 wt. %) were prepared by manually mixing and grinding Mn_55_Bi_45_ and Fe_70_Si_10_B_20_ powders in an agate mortar. This method is considered a viable soft approach for producing a homogeneous powder mixture to be used in various sintering processes^37^, while energetic methods such as ball milling might introduce into the MnBi lattice undesirable defects which can accelerate the decomposition of the LTP phase^10,38^. The mixture was poured into a graphite die (1 cm diameter) and a disk was sintered by SPS (FCT System GmbH—HP D 5 SPS furnace). Processing was performed in vacuum (initial pressure of 40 Pa) at 200 °C with a dwell time of 2 min under a uniaxial pressure of 92 MPa. The heating rate was 100 °C/min and the sample was cooled by cutting the power of the SPS furnace. SPS temperature was selected taking into account that at 200 °C decomposition of LTP is minimized, whereas sintering is active^20^.

A Bruker-AXS D8 ADVANCE diffractometer (Cu _K*α*1_ radiation, *λ* = 1*.*5406 Å) was used to measure X-ray diffraction (XRD) patterns at room temperature (RT). The Rietveld refinement was done by Materials Analysis Using Diffraction (MAUD) software version 2.98. The reported values for the crystallite size and microstrain were obtained via a specific algorithm. The standard reference material 1976b was used for the fit of instrumental function parameters.

Scanning Electron Microscopy (SEM) observations and Energy-Dispersive X-ray spectroscopy (EDS) elemental analyses were performed with a TESCAN Lyra3 XMU Dual Beam, equipped with a Field Emission Gun.

Transmission Electron Microscopy (TEM) investigations were carried out with the JEOL 2100 instrument, equipped with an Energy-Dispersive X-Ray Detector (JEOL). The specimens were prepared by manually crushing the sample in the mortar, dispersing the resulting material in hexane environment followed by drop-casting the resulting suspension on a standard Cu grid (lacey C, 400 mesh).

For Mössbauer spectroscopy studies we used a conventional spectrometer with a ^57^Co (Rh matrix) source, mounted on a drive unit working under the constant acceleration mode (triangular waveform). Powder samples were introduced in a He closed-cycle cryostat. Spectra were acquired in transmission geometry at 300 K and 6 K. The NORMOS computer program was used for fitting the Mössbauer spectra via hyperfine magnetic field distribution components^39^. The isomer shifts are reported relative to α-Fe at room temperature.

Magnetization curves versus magnetic field, *m*(*H*), or temperature, *m*(*T*), were measured with a VSM option on a PPMS machine (DynaCool PPMS-9T, Quantum Design, US).

Samples were labeled as follows: ‘MnBi pwd’ denoting the MnBi raw powder, and MnBi-0, MnBi-3, MnBi-5, MnBi-10, MnBi-20 denoting sintered bulks, where the numbers represent the weight percentages *x* of Fe–Si–B in samples Mn_55_Bi_45_ + *x*⋅Fe_70_Si_10_B_20_, where *x* = 0, 3, 5, 10, 20 wt.%.

## Results and discussion

### Characterization of the MnBi raw powder and pristine sintered sample

**Figure 1:**
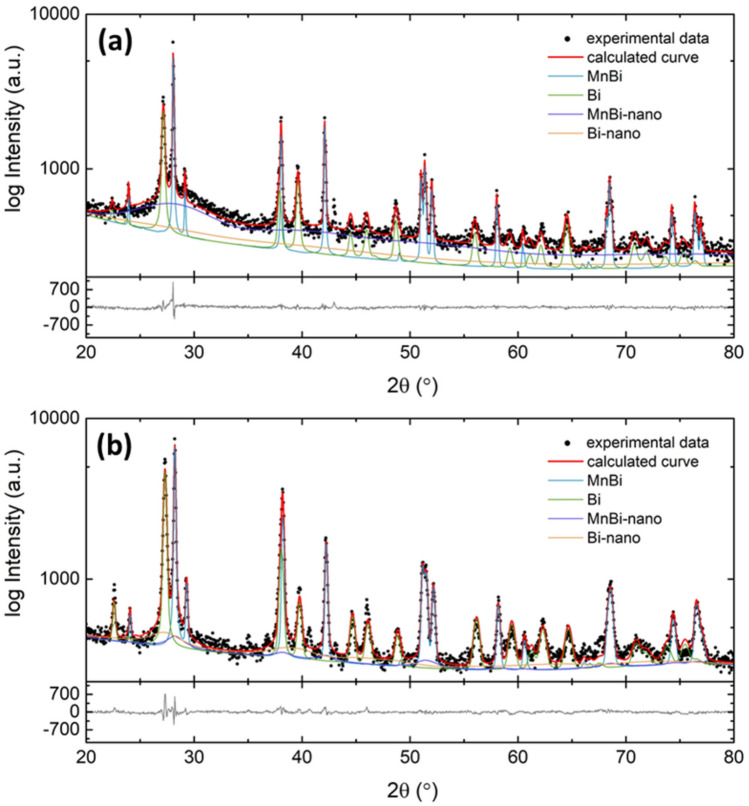
XRD patterns and Rietveld refinements of MnBi-pwd (**a**) and MnBi-0 (**b**). The main phases correspond to the following files: ICDD-04–003-1943(MnBi), ICDD-00–044-1246(Bi).

Figure 1 presents RT XRD patterns of ‘MnBi pwd’ and MnBi-0 sintered bulk. The constituent phases with their relative amount (in wt. %) as provided by the Rietveld refinements are displayed in Table 1. In both samples, the diffraction peaks corresponding to the LTP-MnBi hexagonal structure are dominant. The less intense diffraction peaks assigned to elemental Bi show that Bi is a residual (minor) phase. The specific shape of the peaks with notable basal line-broadening evidence distinct nanostructured phases, specific to both MnBi and Bi. These additional phases, namely Bi-nano and MnBi-nano represent nanostructured phases with very low crystalline coherence length. Herein, the MnBi-nano and Bi-nano have the lattice constants equal to those refined for the crystalline phases MnBi, and respectively Bi, but with the values of crystallite size in the order of nanometers. To properly fit the basal line-broadening, the consideration of the nanostructured phases was required. SPS promotes the formation of more Bi (from 13.7 wt.% in the raw MnBi powder to 32 wt.% in sintered MnBi-0) suggesting modification of MnBi and release of elemental Bi. Apparently, in the sintered sample, there is an enhanced crystallization of MnBi nanograins with the precipitation of large-size Bi crystallites. The RT XRD patterns of both samples did not show any peaks of Mn or Mn oxide. The average crystallite size of MnBi decreases during SPS, thus supporting the proposed scenario. Lattice parameters of MnBi do not change during SPS (see Table 1).

**Table 1:** The constituent phases and relative content, R, of ‘MnBi pwd’ and MnBi-0 as provided by the Rietveld refinements. Lattice parameters, crystal size, ${D}_{eff}$ , and lattice strain, ${\langle{\varepsilon }^{2}\rangle}^\frac{1}{2}$ , are also given.

Phase Sample	MnBi	Bi	MnBi-nano	Bi-nano
MnBi pwd	R = 22.3 wt.% *a* = 4.290 Å *c* = 6.121 Å ${D}_{eff}$ = 310 nm ${\langle{\varepsilon }^{2}\rangle}^\frac{1}{2}$= 6⋅10^–4^	R = 13.7 wt.% *a* = 4.547 Å *c* = 11.888 Å ${D}_{eff}$ = 64 nm ${\langle{\varepsilon }^{2}\rangle}^\frac{1}{2}$= 2⋅10^–3^	R = 52.3 wt.%	R = 11.7 wt.%
MnBi-0	R = 51.3 wt.% *a* = 4.291 Å *c* = 6.120 Å ${D}_{eff}$ = 159 nm ${\langle{\varepsilon }^{2}\rangle}^\frac{1}{2}$= 1.4⋅10^–3^	R = 32 wt.% *a* = 4.546 Å *c* = 11.874 Å ${D}_{eff}$ = 98 nm ${\langle{\varepsilon }^{2}\rangle}^\frac{1}{2}$ = 3.3⋅10^–3^	R = 2.3 wt.%	R = 14.4 wt.%

**Figure 2:**
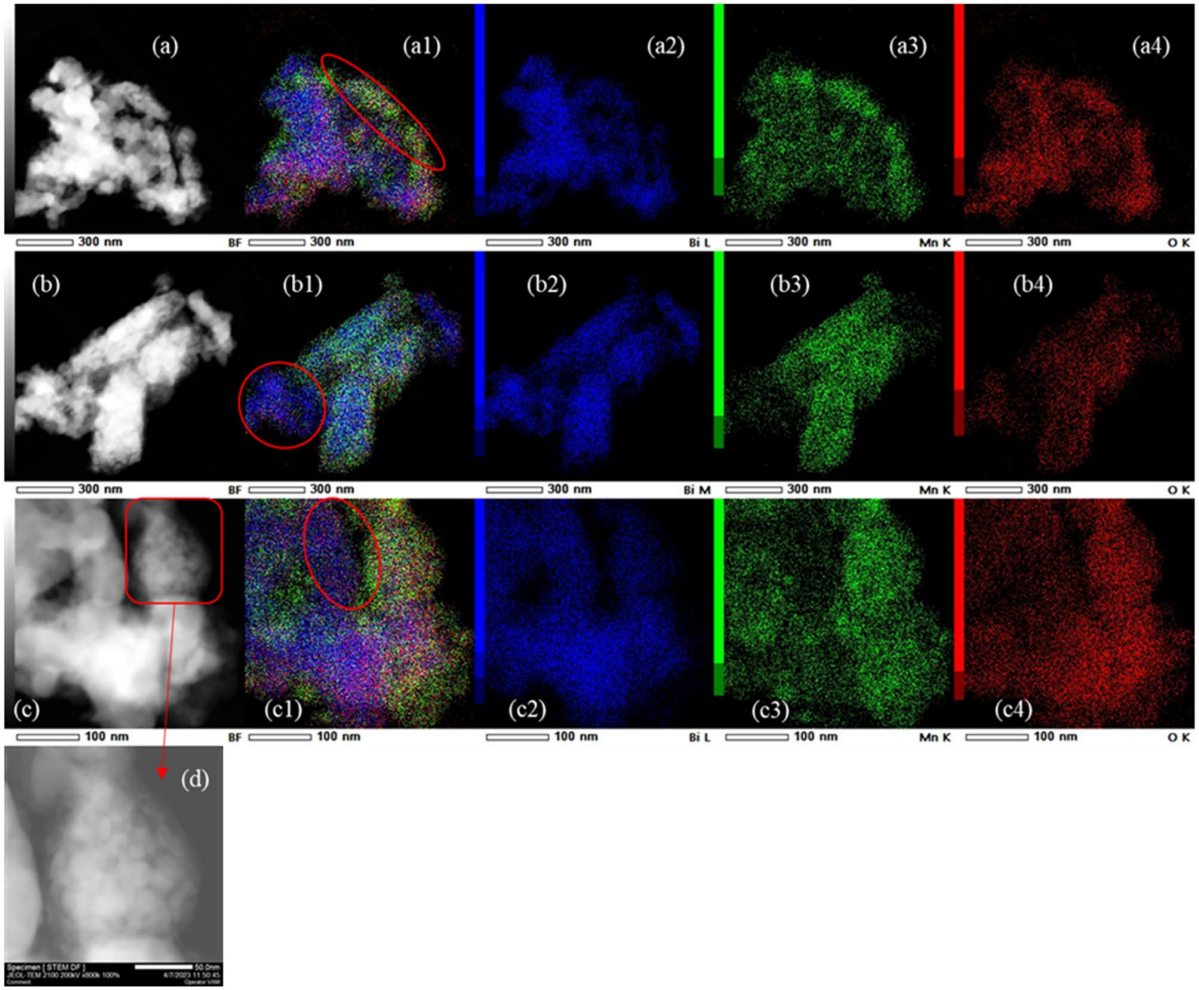
TEM-EDS data of ‘MnBi pwd’ sample: (**a–c**) TEM images; (**d**) TEM image detail of (**c**) at high magnification; (**a1–c1**) red–green–blue (RGB) images obtained by overlapping EDS maps of (**a2–c2**) for Bi, (**a3–c3**) for Mn, and (**a4–c4**) for O. Mn oxidation (**a1**) and Bi segregation (**b1,c1**) regions are indicated by red ellipses.

Elemental maps measured in TEM on the ‘MnBi pwd’ sample are presented in Fig. 2. The elemental distribution is non-uniform and apart from regions ascribed to MnBi phase, one can observe Bi (b1, c1), and MnO (a1) areas. Data also reveal oxidation at the surface of some particles from the investigated agglomerate/grain, especially those being Mn-rich (a1). A detail (d) on image (c) indicates the presence of a fine nanostructure composed of nanoparticles of less than ~ 25 nm. These nanoparticles with a plate- or flake-like hexagonal appearance contain Mn and Bi and are ascribed to the MnBi phase. The elemental non-homogeneity is also inferred from the ratio between the atomic concentrations of Mn and Bi measured on different regions (a), (b), and (c) which spread within a relatively large range, from 0.5 to 1.65 (Fig. 2 and Table 2).

**Table 2:** The average elemental EDS composition and Mn/Bi ratio for regions (a)–(c) presented in Fig. 2.

Maps on	Element	Atomic %	Mn/Bi ratio
Figure 2a	O	54.12	1.65
	Mn	28.58	
	Bi	17.3	
Figure 2b	O	60.02	0.5
	Mn	13.41	
	Bi	26.57	
Figure 2c	O	62.8	0.63
	Mn	14.4	
	Bi	22.8	

**Figure 3:**
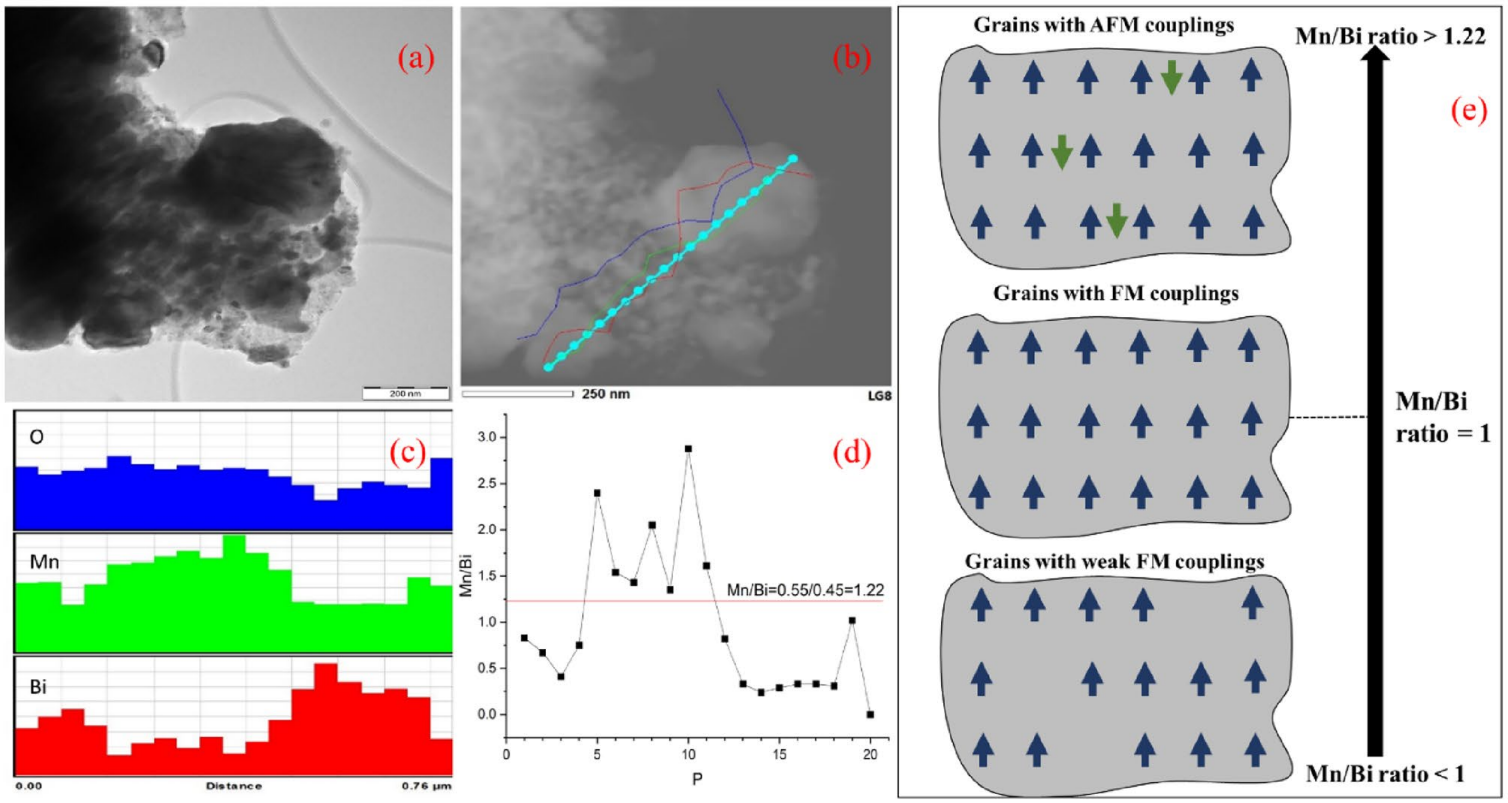
(**a**) TEM image of SPSed MnBi-0 and (**b**) EDS elemental composition measured along the indicated line; (**c**) histograms of specific elements (Mn, Bi, and O) showing variation of composition along the EDS line in (**b**); (**d**) Mn/Bi ratio determined along the EDS composition line in (**b**) and using data from the histogram (**d**). In (**d**), the *x*-axis P shows the point number on the EDS compositional line from (**b**), where P = 1 is the point on the left side and P = 20 is the point on the right side in (**b**). The designed atomic ratio Mn/Bi = 55/45 = 1.22, which approaches the average composition along the investigated micrometer size region, is indicated; (**e**) schematic representation of Mn spins in Mn–Bi grains with MnBi ratio crossing the value of 1, i.e., for Mn/Bi ratio lower than 1, weak ferromagnetic interactions are involved, whereas for Mn/Bi ratio higher than 1, a progressively increasing number of antiferromagnetic (AFM) interactions are involved.

In the TEM image of MnBi-0 sintered sample (Fig. 3a) are present grains with flake-like morphology and some of them are large, of 250 nm or more. An EDS line profile analysis (cyan line in Fig. 3b) reveals a non-uniform Mn, Bi, and O distribution (Fig. 3c, d) that was also observed in the initial raw powder (MnBi pwd). The associated compositional histograms in Fig. 3c and the extracted Mn/Bi ratio in Fig. 3d present an even larger scattering (0.33–2.9) compared to ‘MnBi pwd’ (0.5–1.65). This is expected to negatively influence the magnetic properties of the bulk. Li et al.^40^ suggested that during SPS there is an inhomogeneous distribution of temperature in the grain, i.e. the temperature increases from the particle-contacting surface to the center when the pulsed current passes through. This process may promote Bi precipitation and can explain the large spatial compositional scattering. It is worth to note that this situation is not compatible with the ‘Bi grain boundary phase’ from literature where the Bi-phase separates MnBi crystal grains. According to^41^ this kind of Bi grain boundary phase microstructure promotes enhancement of the hard magnetic properties of the MnBi bulk (a decreased saturation magnetization and an increased coercivity). In ref.^21^ magnetic isolation was obtained through a grain boundary phase containing Mn, Bi, and O. This second type of precipitation promotes an increased magnetization and a decreased coercive field. According to Table 1, an increased amount of Bi precipitates is observed in sample MnBi-0 (~ 46 wt.%) as compared with sample MnBi pwd (~ 25 wt.%). As will be shown later, an increased saturation magnetization and a decreased coercive field are observed in the sample with a higher amount of Bi precipitation (MnBi-0). Therefore, this second type of Bi precipitation at the grain boundaries seems to comply with our results.

This type of microstructure promoting inter-granular precipitation was considered suitable for introduction of soft magnetic additives (FeSiB in this work) at grain boundaries to target hard-soft magnetic interactions. For this aim, a powder solid state route by using soft magnetic additives instead of melting the powder mixture as usually reported in the literature can be rewarding and justifies our choice of technological approach for the fabrication of MnBi–FeSiB bulk composite magnets.

**Figure 4:**
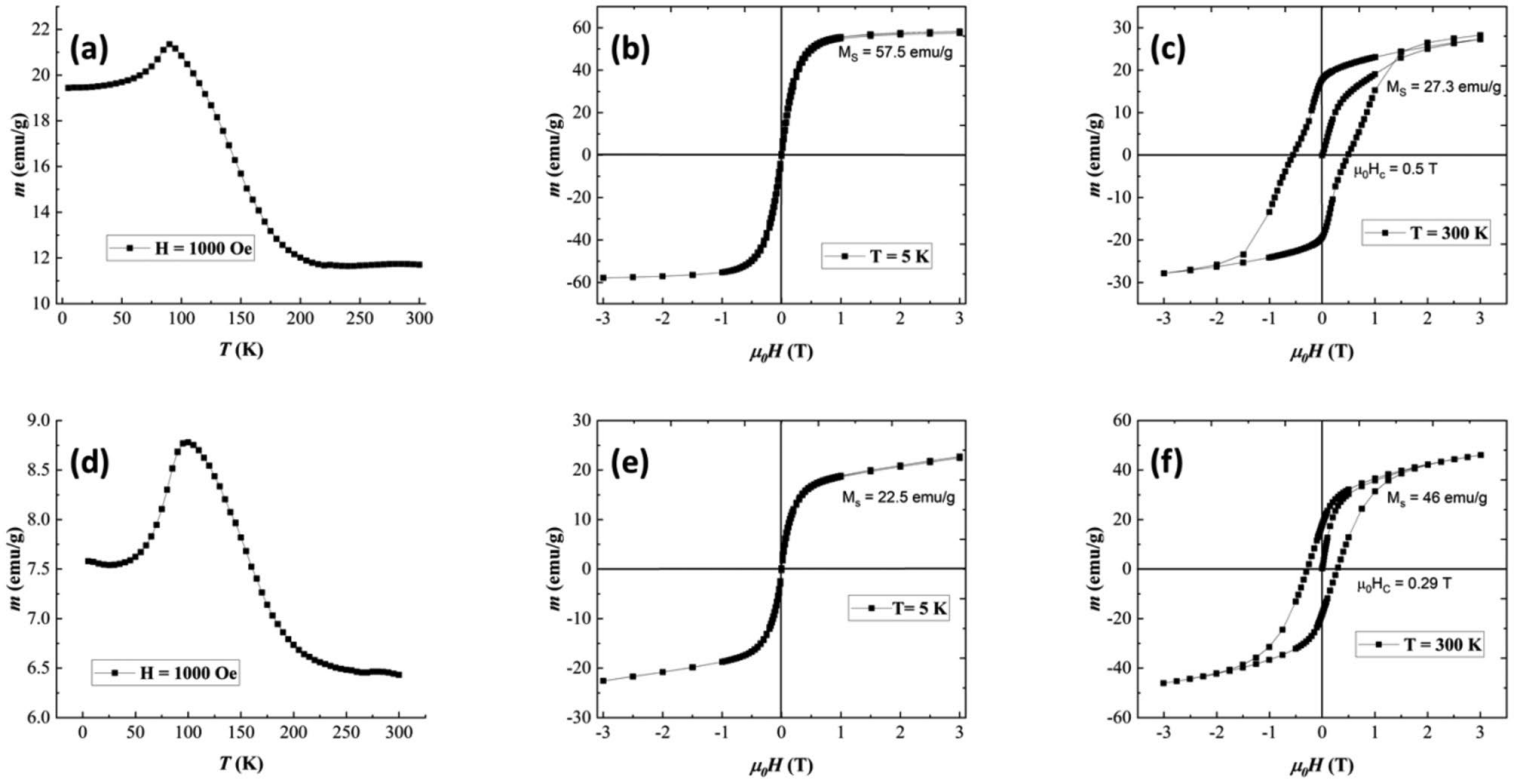
Thermomagnetic curve for ‘MnBi pwd’ under a magnetic field of 1000 Oe (**a**) and associated hysteresis loops at 5 K (**b**) and 300 K (**c**). The thermomagnetic curve for MnBi-0 under a magnetic field of 1000 Oe (**d**) and associated hysteresis loops at 5 K (**e**) and 300 K (**f**).

Temperature-dependent magnetization (Fig. 4a, d) in 1000 Oe suggests spin reorientation above 90 K for both ‘MnBi pwd’ and MnBi-0 samples. This can be attributed to the change of planar anisotropy (*ab* plane) at low temperature into a uniaxial orientation (along the *c axis*) above 90 K for the Mn spins^42^, giving rise to the local maximum in *m*(*T*) curve in Fig. 4a, d. Accordingly, the LTP MnBi presents negative anisotropy al low temperature. It increases with temperature going through zero at about 100 K reaching the highest positive value at about 490 K^43^: there is this temperature close to 100 K, where due to the low anisotropy constant the magnetic moments can easily orient along the applied field, giving rise to the local increase of magnetization. Except for this behavior, it is worth mentioning the substantial decrease of magnetization at high temperatures, ending up with a plateau above about 200 K. This plateau shows that the decrease of magnetization cannot be related to only magnetic relaxation phenomena at temperatures approaching the Curie temperature, and an additional magnetic effect should be considered. It is noteworthy, that theoretical and experimental studies have proven almost negligible magnetic moment of Bi in both MnBi compounds or clusterized metallic Bi^44–47^. Therefore, only the Mn magnetic moments will be considered in the following assumptions. Previous studies reported a complex magnetic structure correlated to the Mn–Mn atomic distance and how it influences the spin ordering^43,48^. It has been proven that contrary to the ferromagnetic (FM) exchange interaction between the Mn spins in the case of the well-formed equiatomic LTP MnBi, the interstitial occupation of the Mn atoms leads to an antiferromagnetic (AFM) interaction between the Mn neighbors. While in the experimentally stable compound (e.g. the Mn_55_Bi_45_ as we consider here), there is always a certain occupation of interstitial sites by the Mn atoms, it means that in this real compound one deals always with competing FM (dominant), and AFM interactions. Having in mind the volumetric distribution of the Mn content (see e.g. Fig. 3d), one can see that there will be crystallites with a much higher Mn content (Mn/Bi ratio much higher than 1.22), and hence with an increased number of interstitial Mn (i.e. with a relatively increased number of AFM interactions, Fig. 3e). There are also crystallites with a much lower Mn content (Mn/Bi ratio lower than 1), i.e. with a lack of interstitial number of Mn atoms (i.e. presenting only FM interactions). Accordingly, there will be crystallites characterized by a higher and lower magnetization and the higher is the density of interstitial Mn atoms, the lower will be the total average magnetization. On the other hand, although crystallites with a lower Mn content present only FM interactions and, hence, magnetization is higher, their specific exchange integrals are much weaker due to the low density of magnetic centers (Mn atoms). As a consequence, the FM interactions in such crystallites of low Mn content can be destroyed at much lower temperatures than the usual Curie temperature of a perfect Mn_55_Bi_45_ compound (with Mn/Bi ratio equating to 1.22 everywhere in space). Accordingly, when the temperature increases, one deals with a broad magnetic relaxation, the faster one belonging to the crystallites of lower Mn content. It is this additional faster magnetic relaxation in crystallites presenting FM ordering at low temperatures, which leads to the decrease of the overall magnetization above 200 K. In addition, the specific step-like shape of the magnetization decrease versus temperature suggests a rather bimodal distribution of the Mn content in the crystallites, which is also roughly supported by the evolution of the Mn/Bi ratio shown in Fig. 3d.

At 5 K (Fig. 4b, e), the saturation magnetization *M*_*S*_ = 57.5 Am^2^ kg^−1^ (57.5 emu g^−1^) in the ‘MnBi pwd’ is more than two times higher than MnBi-0, with *M*_*S*_ = 22.5 Am^2 ^kg^−1^ (22.5 emu g^−1^). Looking to the XRD results in Table 1 for the two samples, one observes an increased amount of Bi clusters in the MnBi-0 sample (e.g. about 46 wt.% as compared to about 25 wt.% in ‘MnBi pwd’). Consequently, the remaining LTP MnBi in the BiMn-0 sample should be characterized by an increased Mn content and also by an increased density of interstitial Mn atoms in the crystallites. That is, an increased density of AFM couplings leading to a decreased saturation magnetization is expected at low temperatures in the MnBi-0 sample as compared to ‘MnBi pwd’. On the other hand, a lower density of crystallites with FM ordering is expected in this sample and, as a consequence, a lower step-like decrease of magnetization above 200 K occurs (see Fig. 4a, d). When increasing the temperature at 300 K from 5 K, two mechanisms are activated: (i) an enhanced magnetic relaxation in the grains with weak FM couplings and (ii) a cut-off of AFM couplings in the grains with AFM coupling. So, many pairs of Mn atoms with AFM coupling (without contribution to the magnetization at low temperature) will become paramagnetic at 300 K and in high magnetic fields will give additional contribution to the saturation magnetization. From the interplay between the two mechanisms, the overall saturation magnetization can either decrease with temperature (in sample MnBi-pwd with a relative higher number of grains with weak FM couplings) or increase (in sample MnBi-0 with a relative higher number of grains with AFM couplings). By looking at the hysteresis loops of MnBi pwd and MnBi-0 measured at 300 K Fig. 4c, f, a kink is observed in the magnetization reversal curve of ‘MnBi pwd’. This can be attributed to the different coercivities resulting from the two types of LTP MnBi, well crystallized and nano. Due to the small amount of MnBi-nano phase in the sample (about 3%), there is an absence of a kink on the 300 K data collected for MnBi-0. The much higher overall coercivity in ‘MnBi pwd’ has to be related also to the higher amount of MnBi nanophase in this sample (e.g. about 52% as compared with about 3% in sample MnBi-0, Table 1).

Another specificity of the loops collected at 300 K (Fig. 4c, f) is the incomplete saturation even in applied fields of 3 T. That is in agreement with the above assumption that at 300 K there are specific MnBi crystallites of lower Mn content, which behave paramagnetically above 200 K. This paramagnetic component leads to the almost linear increase of magnetization in high fields with a slope proportional to the number of paramagnetic centers.

The coercive field has the expected tremendous increase with the temperature, as specific to the MnBi compound displaying a positive temperature coefficient of coercivity. The increase of *μ*_*0*_*H*_*C*_ is from almost 0 T at 5 K to 0.50 T and 0.29 T at 300 K in ‘MnBi pwd’ and MnBi-0, respectively. However, the morphology and grain size strongly influence the coercivity and the lower value in the MnBi-0 sample can be attributed to the misalignment of the grains and their growth mechanism during SPS. This sample (MnBi-0) of low coercivity is also characterized by a relatively low amount of the phase MnBi-nano (Table 1).

### Characterization of sintered MnBi–FeSiB nanocomposites

**Figure 5:**
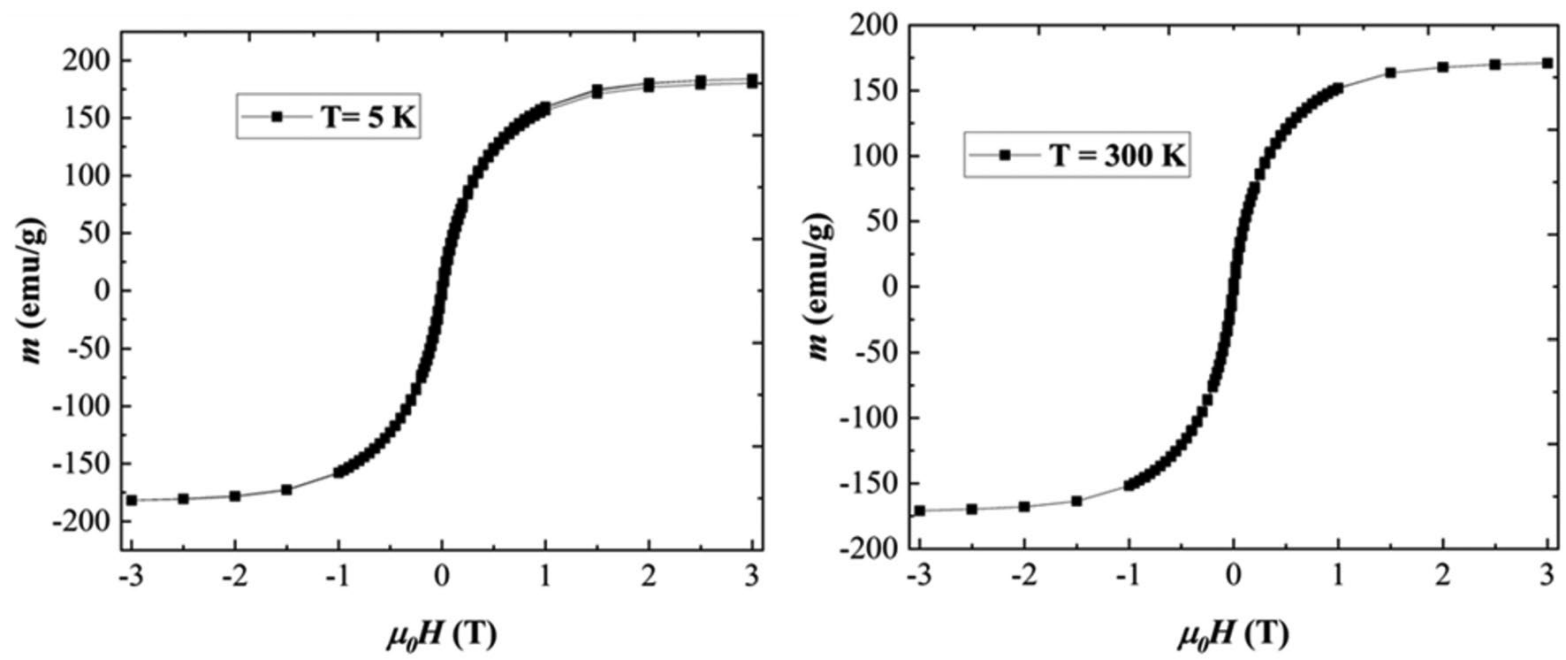
Hysteresis loops at 5 K (**a**) and 300 K (**b**) of Fe_70_Si_10_B_20_ ribbons.

To determine the magnetic properties of the soft magnetic phase, hysteresis loops were collected at 5 K and 300 K on the amorphous FeSiB ribbons (Fig. 5a, b). Coercivity *H*_*c*_ is small and it decreases from 68 Oe (0.0068 T) at 5 K to 38 Oe (0.0038 T) at 300 K. The saturation magnetization at 5 K is high i.e., 183.9 Am^2^ kg^−1^ (183.9 emu/g) and it decreases slightly at 300 K to 170.87 Am^2^ kg^−1^ (170.87 emu/g). Both these parameters confirm that indeed the FeSiB ribbons represent a convenient soft magnetic material of high magnetization and low coercive fields, promoting them as a suitable additive for the MnBi-based nanocomposites.

**Figure 6:**
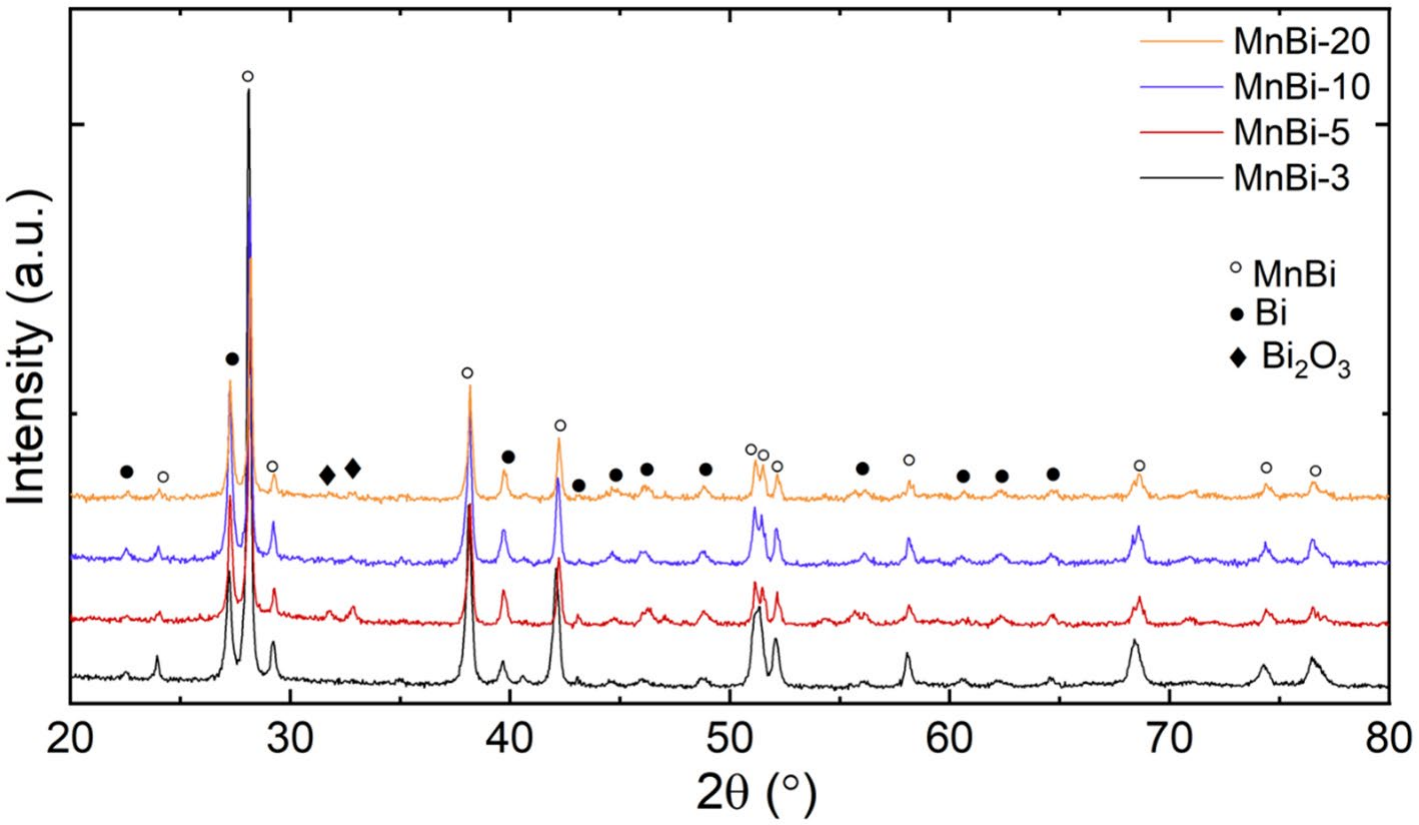
XRD patterns of MnBi-3 (black), MnBi-5 (red), MnBi-10 (blue), and MnBi-20 (yellow). The main phases correspond to the following files: ICDD-04-003-1943(MnBi), ICDD-04-007-1443(Bi_2_O_3_), ICDD-00-044-1246(Bi).

The room temperature XRD patterns of the MnBi-FeSiB nanocomposites and their relative phase content extracted from Rietveld analysis are presented in Fig. 6 and Table 3. The main phases are LTP-MnBi and MnBi-nano and the secondary phases are Bi and Bi_2_O_3_. Due to low content and amorphous state, FeSiB additive provides no observable signal in XRD patterns: clusters of elemental Fe, Si, B, or Mn were not detected in the diffraction patterns. When FeSiB is added, the variation of lattice parameters of the main phases is limited. This may suggest that there is no substitutional or interstitial site occupancy in the crystal lattice of LTP MnBi (see Table 3). However, a slight recrystallization of FeSiB was observed by Mӧssbauer spectroscopy, as discussed in the next paragraphs. One also observes that the introduction of FeSiB in MnBi samples with *x* = 3, 5, 10, 20 wt.% diminishes the decomposition of MnBi phases as compared to sample MnBi-0, i.e. the overall amount of Bi or Bi_2_O_3_ is lower in the composite samples. The main two consequences of the FeSiB additive are (1) reducing the amount of Bi and (2) the presence of more MnBi nanophase in the composite sintered samples. Remarkably, a quite similar effect where carbon helps to decrease the amount of Bi in Mn_55_Bi_45_ was also observed by Kharel et al. in ref.^22^.

**Table 3:** The constituent phases and their relative content of MnBi-*x* (where *x* = 3, 5, 10, 20 wt. % of FeSiB) as provided by the Rietveld refinements.

Phase Sample	MnBi	Bi	MnBi-nano	Bi_2_O_3_
MnBi-3	R = 79.9 wt. % *a* = 4.292 Å *c* = 6.112 Å ${D}_{eff}$ = 144 nm ${\langle{\varepsilon }^{2}\rangle}^\frac{1}{2}$ = 2⋅10^–3^	R = 14 wt. % *a* = 4.544 Å *c* = 11.867 Å ${D}_{eff}$ = 61 nm ${\langle{\varepsilon }^{2}\rangle}^{1/2}$ = 2.3⋅10^–3^	R = 6.1 wt. %	–
MnBi-5	R = 43.1 wt. % *a* = 4.289 Å *c* = 6.12 Å ${D}_{eff}$ = 143 nm ${\langle{\varepsilon }^{2}\rangle}^\frac{1}{2}$= 6⋅10^–4^	R = 17.9 wt. % *a* = 4.546 Å *c* = 11.868 Å ${D}_{eff}$ = 73 nm ${\langle{\varepsilon }^{2}\rangle}^\frac{1}{2}$ = 1.1⋅10^–3^	R = 25.2 wt. %	R = 13.8 wt. % *a* = 7.740 Å *c* = 5.641 Å ${D}_{eff}$ = 86 nm ${\langle{\varepsilon }^{2}\rangle}^{1/2}$ = 2.2⋅10^–3^
MnBi-10	R = 61.6 wt.% *a* = 4.289 Å *c* = 6.119 Å ${D}_{eff}$ = 147 nm ${\langle{\varepsilon }^{2}\rangle}^\frac{1}{2}$ = 8⋅10^–4^	R = 26.3 wt. % *a* = 4.544 Å *c* = 11.868 Å ${D}_{eff}$ = 75 nm ${\langle{\varepsilon }^{2}\rangle}^\frac{1}{2}$= 2⋅10^–3^	R = 12.1 wt.%	–
MnBi-20	R = 50 wt. % *a* = 4.288 Å *c* = 6.119 Å ${D}_{eff}$ = 166 nm ${\langle{\varepsilon }^{2}\rangle}^\frac{1}{2}$ = 7⋅10^–4^	R = 23.3 wt. % *a* = 4.545 Å *c* = 11.86 Å ${D}_{eff}$ = 76 nm ${\langle{\varepsilon }^{2}\rangle}^\frac{1}{2}$= 1.5⋅10^–3^	R = 19.2 wt.%	R = 7.5 wt. % *a* = 7.744 Å *c* = 5.646 Å ${D}_{eff}$ = 78 nm ${\langle{\varepsilon }^{2}\rangle}^\frac{1}{2}$= 1.5⋅10^–3^

Lattice parameters, crystal size, ${D}_{eff}$ , and lattice strain, ${\langle{\varepsilon }^{2}\rangle}^\frac{1}{2}$, are also given.

**Figure 7:**
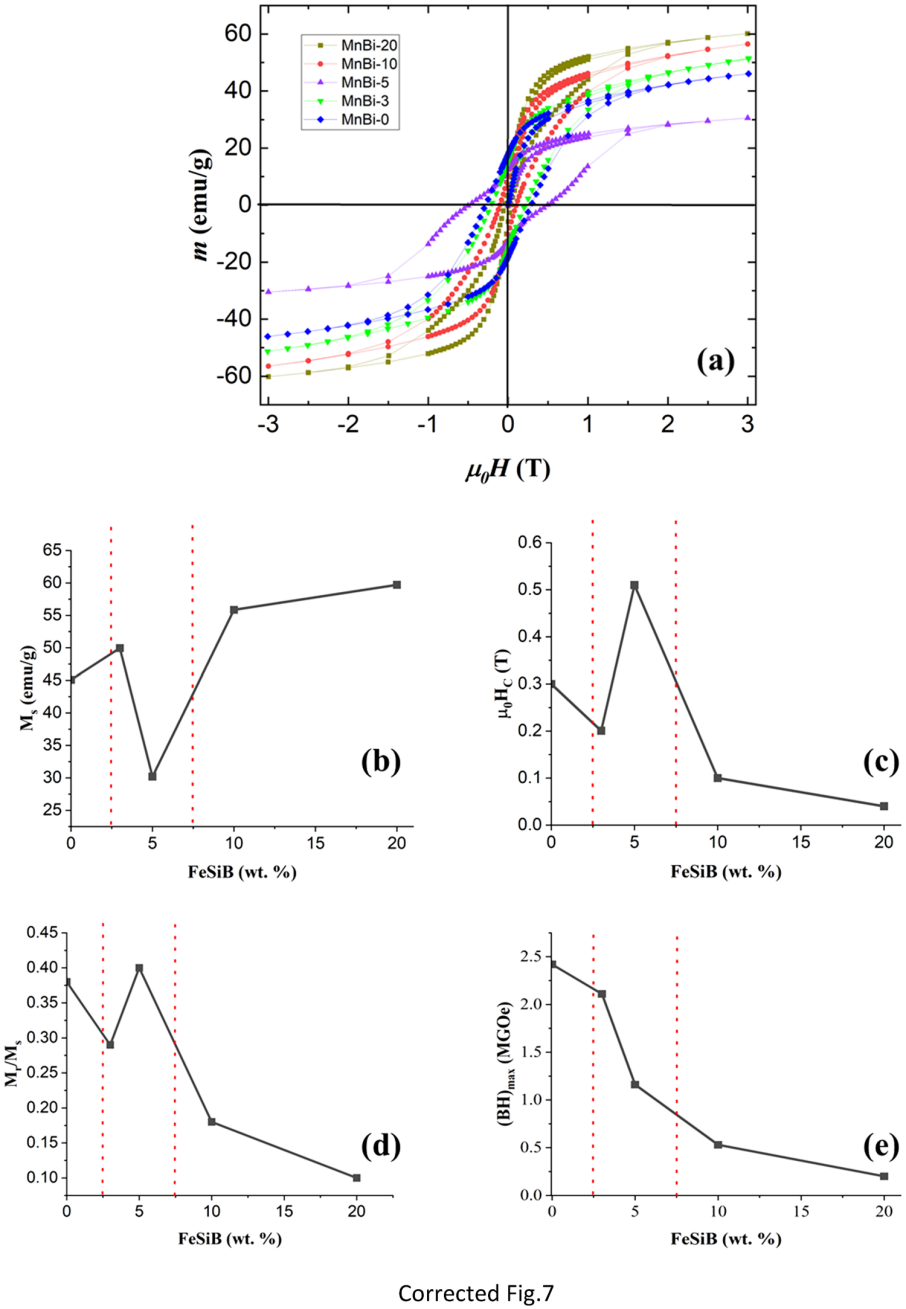
*m*(*H*) magnetic loops collected at room temperature for a magnetic field of 3 T (**a**), saturation magnetization (**b**), coercitive field (**c**), remnant ratio (**d**) and maximum energy product (**e**) of MnBi samples with different weight percentages of FeSiB addition.

Figure 7a shows the hysteresis loops of the bulk sintered samples, recorded at RT. Although some samples are not saturated completely at 3 T, the saturation values are calculated using the law of approach to saturation^49^. By increasing the FeSiB addition, the saturation magnetization is increasing for MnBi-3, MnBi-10, and MnBi-20 (49.96 emu g^−1^, 55.85 emu g^−1^ and 59.71 emu g^−1^, respectively, see Fig. 7b) compared to the corresponding magnetization of MnBi-0 (46 emu g^−1^). This is connected, on one hand, to the high saturation magnetization of the FeSiB, and, on the other hand, to a lower decomposition of the LTP MnBi in the composite samples, following the relative phase content presented in Table 3. On the contrary, with the *M*_s_ enhancement, *H*_c_ at room temperature decreases with increasing of *x*, as shown in Fig. 7c. The only exception is the enhanced coercivity and decreased magnetization of the MnBi-5 sample. This sample does not follow the general trends of *M*_s_ and *H*_c_ displayed by the other sintered samples. Therefore, first, we shall discuss the samples respecting the general trends (MnBi-3, MnBi-10 and MnBi-20) and after that, we shall consider the peculiar features of sample MnBi-5.

In the case of a simple additive contribution of saturation magnetization of the soft magnetic phase (i.e. of about 170 emu/g), which is valid if exchange spring effects are disregarded, the expected saturation magnetization of the composite samples MnBi-3, MnBi-10, and MnBi-20 would be about 49.7 emu g^−1^, 58.4 emu g^−1^ and 70.8 emu g^−1^. The assumption is valid under the condition that the morpho-structural and compositional characteristics of the hard magnetic MnBi phases would be similar to the hard magnetic phase in the MnBi-0 sample. However, the overall amount of the Bi phases in these composite samples is lower (ranging from 14 to 31 wt.% according to Table 3) as compared to the Bi phases in MnBi-0 sample (about 46 wt.% according to Table 1). Therefore, the fraction of the AFM couplings in the MnBi phases is lower in the composite samples as compared to the MnBi-0 one. Hence, even higher saturation magnetizations would be expected in the composite samples as compared to the ones above reported from the simple additive contribution of the soft magnetic phase. As a consequence, the lower experimental values reported in Fig. 7b for samples MnBi-3, MnBi-10 and MnBi-20 might be due also to exchange spring effect. However, this effect cannot explain such a large decrease of the saturation magnetization as compared with the contribution of the simple addition of the soft and the presence of the hard magnetic phase to the saturation magnetization and an additional effect might be considered. Due to the microstructural complexity of the investigated systems we will compare only sample MnBi-0 and MnBi-20, containing a similar amount of well-structured MnBi phase (about 50%). The amount of the precipitated Bi phase is about 46% in MnBi-0 and 31% in MnBi-20, meaning that the Mn content in the MnBi phase is higher in the first sample. As a consequence, the number of grains with weak FM couplings is higher for sample MnBi-20. At 300 K, the saturation magnetization of this sample has to be consistently lower than that of MnBi-0 due to the magnetic relaxation of the predominant grains with weak FM couplings. This might be a reliable explanation of the lower saturation magnetization in the composite sample MnBi-20 as compared to the expected value resulting from the additive contributions coming from MnBi-0 and soft magnetic phase. The coercive field of the sintered samples (except MnBi-5) decreases continuously (Fig. 7b), in spite of the fact that the relative content of the MnBi nanophase (responsible for the high coercive field, as previously stated) is increasing continuously for samples MnBi-3, MnBi-10 and MnBi-20. Again, one explanation for a coercive field of the composite sample lower than of the hard magnetic component is the interfacial coupling between the soft and hard magnetic phases, leading to the exchange spring effects. As an additional confirmation on the presence of such effects, we have considered the Henkel plot (M plot) representations on some samples^50–52^. Accordingly, the parameter ΔM that provides information on the occurrence and strength of the coupling between the soft and the hard magnetic phases is determined from the M plots via the following relation: ΔM = m_d_(H) − [1 − 2m_r_(H)], where m_r_(H) = M_r_(H)/M_r_(H_max_), m_d_(H) = M_d_(H)/M_r_(H_max_), M_r_(H) is the remnant magnetization obtained on removal of the field H, H_max_ is the maximum applied field for assuring the saturation (3.5 T in the present case) and M_d_(H) is the remnant demagnetization (the remnant magnetization after an increasing negative field is applied, succeeding the initial saturation of the sample in H_max_). An example of M plots with the graphical representation of ΔM versus the applied magnetic field is presented for sample MnBi-3 in Fig. 1 Supplementary material (SM). A positive coupling between the soft magnetic and hard magnetic phases is clearly evidenced in fields of up to 1 T magnetic induction.

Within these results, we bring to attention the complex hysteresis loop of sample MnBi-5 which suggests the presence of 2 magnetic components with different coercivities. Our understanding is that a much weaker exchange spring effect occurs in this sample as compared with the other composite samples MnBi-3, MnBi-10, and MnBi-20. As a consequence, the soft magnetic phase is exchange coupled to only a part of the hard magnetic phase (leading to the magnetic component with a lower coercive field), the rest of the hard magnetic component (of higher coercive field) behaving independently concerning the magnetization reversal process. By looking at Table 3, there are 2 major differences between MnBi-5 and the rest of the samples in the series of sintered composites: (i) the highest amount of MnBi nanophase, i.e. about 25 wt. % and (ii) the highest content of Bi and Bi oxide phases, i.e. of about 32 wt %. Accordingly, the atypically high coercivity of sample MnBi-5 (whereas saturation magnetization is the lowest, Fig. 7a) is explained by the highest amount of uncoupled MnBi nanophase of high coercivity. At the same time, the lowest saturation magnetization of these composite samples could be explained also by the highest amount of AFM couplings among the composite samples, specific to the MnBi LTP. This is due to the highest Mn content in the LTP MnBi uncoupled nanophase, as supported by the enhanced clustering process of the Bi atoms. One notes the almost similar content of Bi and Bi_2_O_3_ phases of about 31 wt. % in sample MnBi-20, but in the last sample the AFM couplings belong to a coupled hard magnetic phase which is strongly interacting with a soft magnetic phase of high magnetization, leading at the end to a higher saturation magnetization of the exchange spring system.

The tendency of the remanence ratio (*M*_r_*/M*_s_) for the sintered samples is shown in Fig. 7d. For MnBi-5 the *M*_r_*/M*_s_ ratio (0.4) is the highest among all samples and it is the closest to the hard magnetic sample MnBi-0. This can be again explained by the significant contribution of the uncoupled hard magnetic MnBi nanophase of high remanent magnetization specific to this system. The energy product *(BH)*_*max*_ was computed as the area of the *m*(*H*) loop. The product defines the figure of merit for any magnetic structure. It reflects the performance of a permanent magnet and is presented in Fig. 7e. The sharpest decrease is for sample MnBi-5 proving again that the exchange spring effects are the weakest in this sample and supporting the idea of a quite different behavior of this sample among the others from the investigated series. Given the minimal decrease of the energy product, sample MnBi-3 shows the strongest exchange spring effects when adding in the MnBi-based composite a relatively small amount of the low-cost and abundant FeSiB soft magnetic component.

Since sample MnBi-5 presents the most intriguing magnetic properties in the series (Fig. 7b–e), next we shall focus on its detailed morpho-structural characterization searching for the intimate reasons for the reduced interfacial exchange spring interaction. A plot with the first derivative of the magnetization versus the applied magnetic field for the magnetic loops presented in Fig. 7a is shown in Supplementary Material Fig. 2 SM, highlighting the two switching fields for MnBi-5 sample.

**Figure 8:**
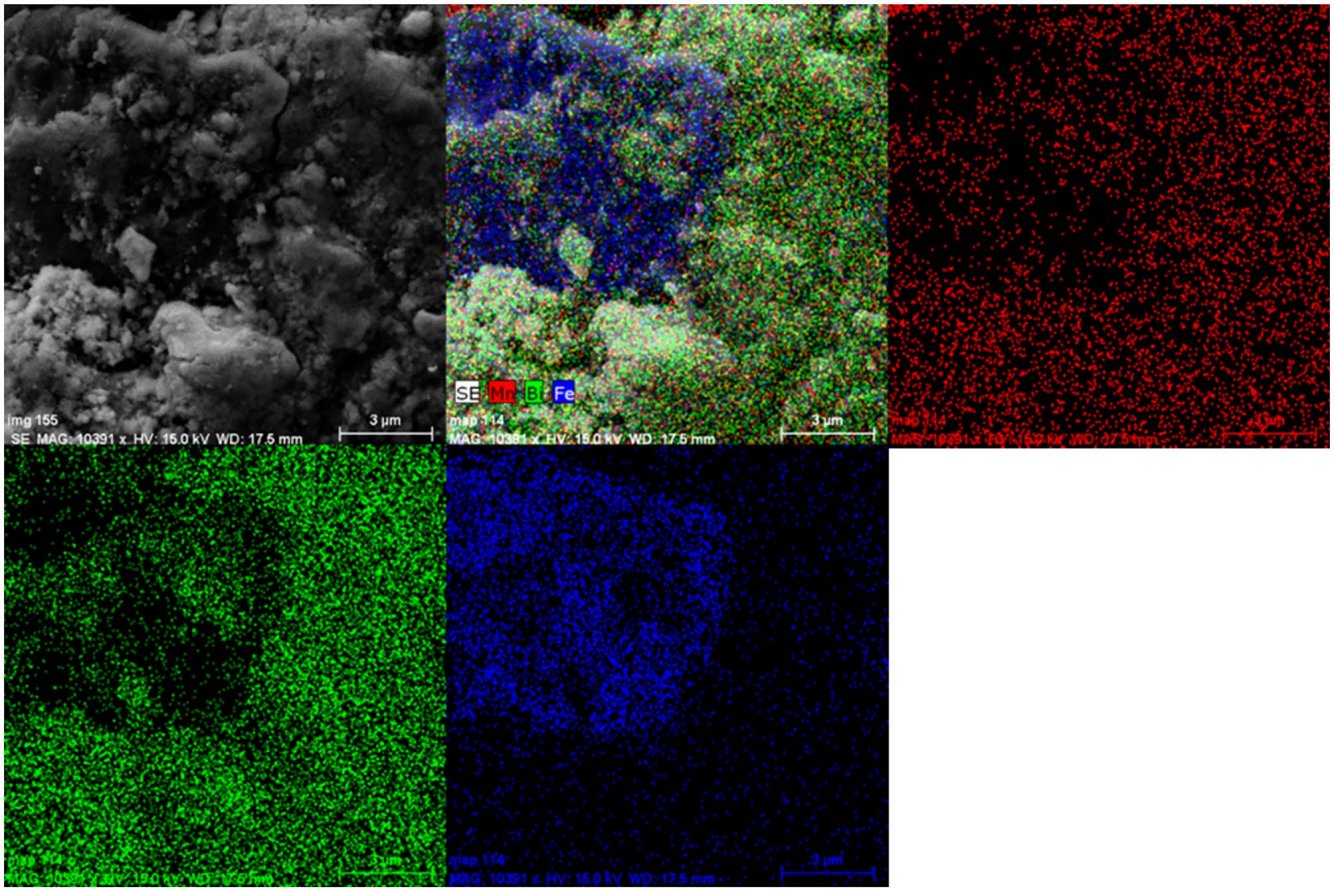
Backscattered SEM image with elemental mapping of MnBi-5 sample.

Figure 8 shows the backscattered SEM images with elemental mapping of the MnBi-5 sample, obtained by EDS. Coarse particles with small flaky grains on their surfaces are observed. The normalized Fe concentration determined from the entire area of photograph from Fig. 8, was determined to be around 40 at.%. This very high value that disagrees with the nominal composition of sample MnBi-5, indicate the sample is not uniform with the photograph here presenting mainly the Fe-rich regions of the soft magnetic phase. Concentrations of Mn and Bi are 34 at.% and 26 at.%, respectively. Thus the ratio Mn/Bi (1.30) is slightly higher than the nominal Mn_55_Bi_45_ composition (with Mn/Bi ratio 1.22). Further, TEM was employed to observe MnBi-5 at higher magnifications.

**Figure 9:**
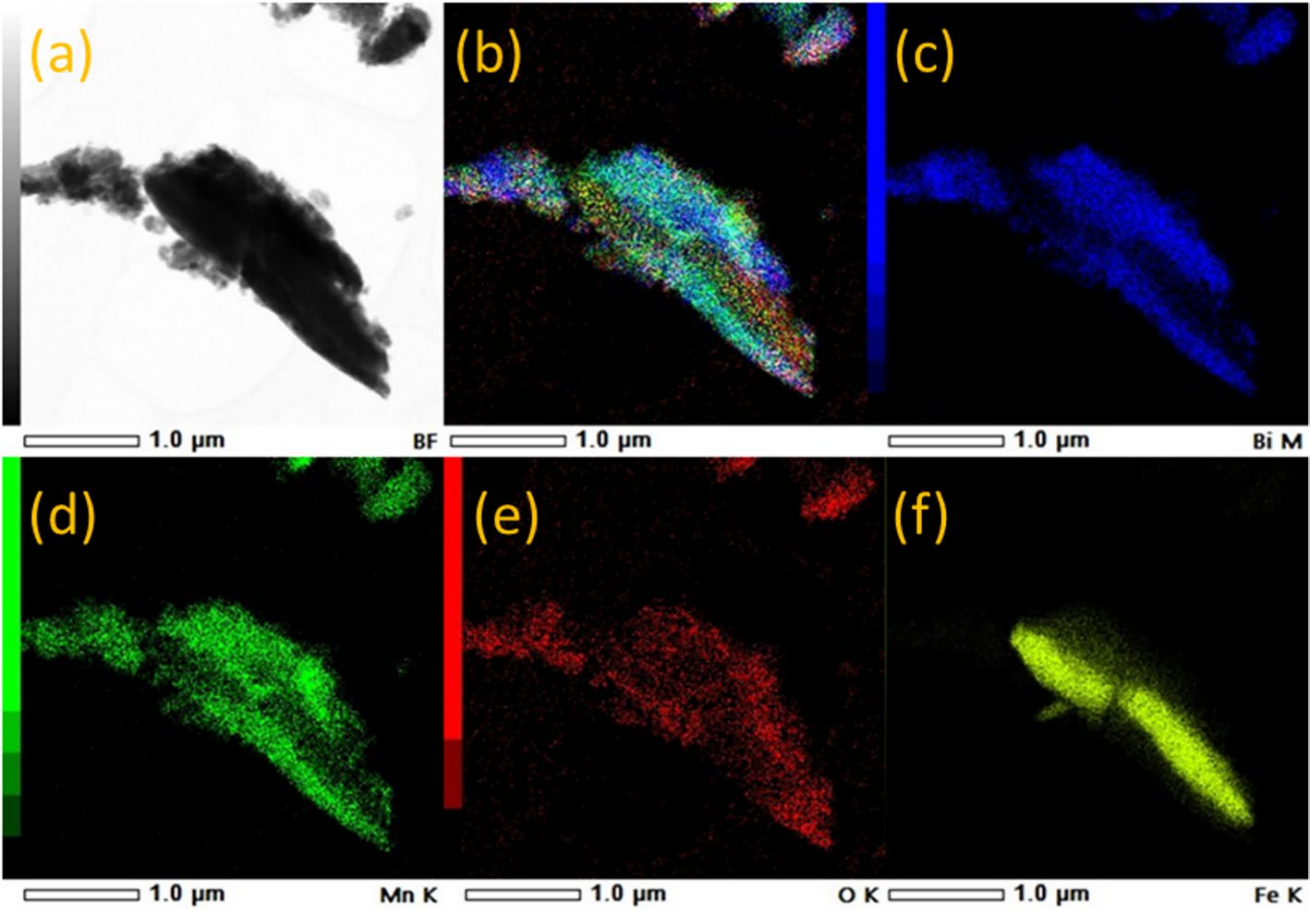
(**a**) TEM image of the sintered MnBi-5 sample, (**b**) RGB image obtained from overlapping EDS compositional maps from (**c–f**).

The TEM image taken on MnBi-5 sample is presented in Fig. 9a. The RGB image presented in Fig. 9b is obtained by overlapping the EDS compositional maps of Bi Fig. 9c, Mn Fig. 9d, O Fig. 9e and Fe Fig. 9f. The Fe-containing area is sandwiched by regions containing Mn and Bi. In these regions, some oxygen is also available. Based on EDS data measured on the entire area, the ratio between the atomic concentrations of Mn and Bi has a value of 1.31. This value is slightly higher than the nominal Mn_55_Bi_55_ composition (Mn/Bi = 1.22) and is in good agreement with the SEM–EDS data presented in Fig. 8. Thus, the elemental analysis confirms the presence of MnBi phases enriched in Mn (with AFM couplings) and their distribution at interfaces with grains of the Fe based soft magnetic phase, as well as the presence of oxygen atoms which might remain at grain boundaries either adsorbed or chemically bounded to atoms of the two involved magnetic phases.

Results from the previous paragraph concerning the magnetic behavior of sample MnBi-5 can be complementary understood by looking on different experiments from literature when using different additives in MnBi. In samples Mn_55_Bi_45-*x*_Ga_*x*_, according to Yang et al.^24^ Ga doping decreases the crystallite sizes. At the same time, in samples Mn_55_Bi_45-*x*_Ga_*x*_ with *x* changing from 1 to 5, *H*_C_ increases. By further increasing Ga concentration up to *x* = 10, *H*_C_ decreases. The improved coercivity at low and increasing *x* values was explained via doping the hard magnetic phase with pinning centers associated to the Ga atoms.

**Figure 10:**
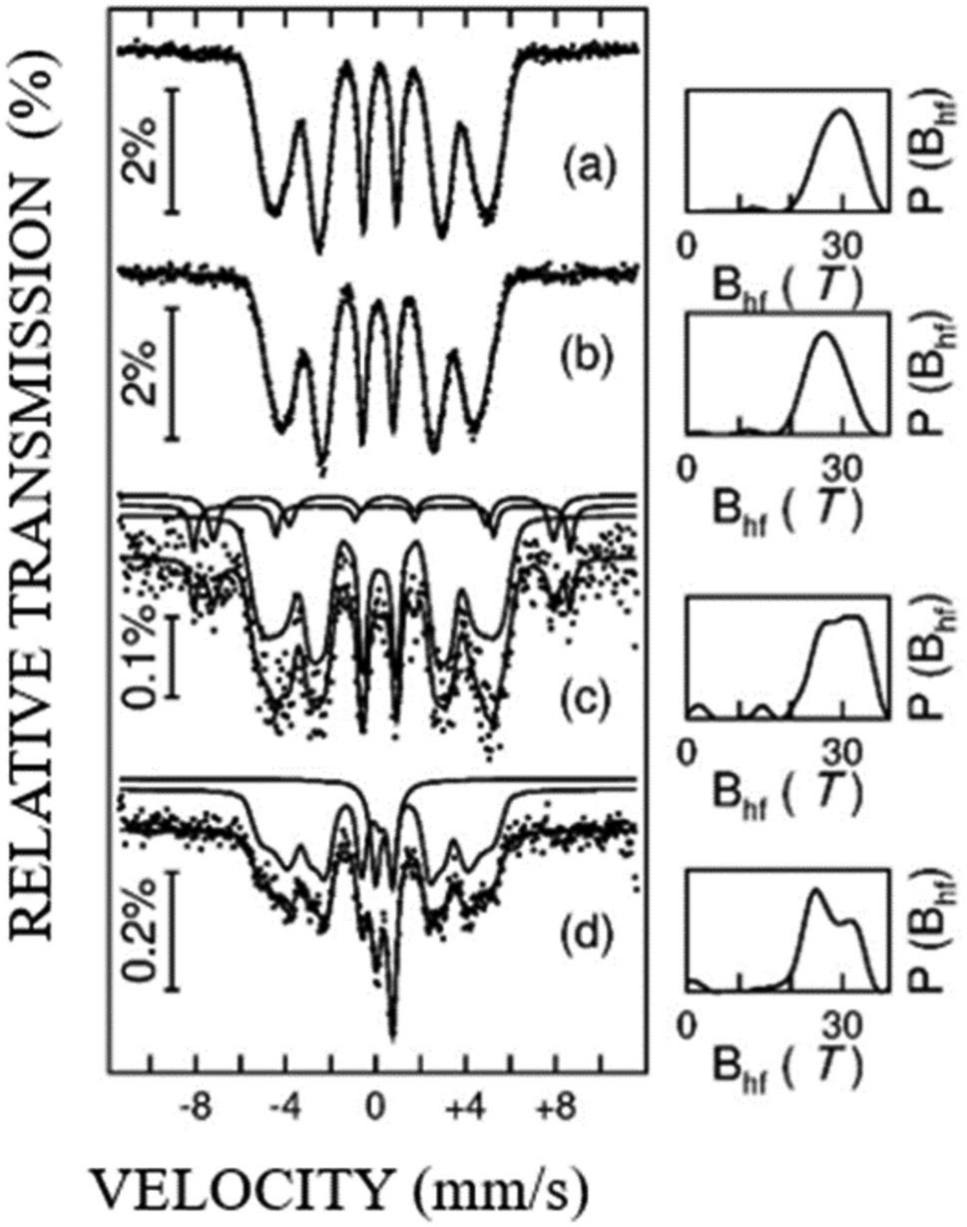
Mössbauer spectra for Fe_70_Si_10_B_20_ collected at two temperatures (5 K (**a**) and 300 K (**b**)) and for MnBi-5 (5 K (**c**) and 300 K (**d**)).

The amount and quality of the LTP-MnBi depend on the doping level of Cr in Mn_50-*x*_Bi_50_Cr_*x*_ (*x* = 0, 1.5, 3, 5) samples, according to ref^53^. There is an enhanced magnetic response by increasing the Cr concentrations up to *x* = 3 followed by a decrease at higher *x*. This was explained by considering a domain wall pinning at the crystallite inhomogeneities. However, in none of these cases, there is an explanation for inverting the trend of coercive field when overpassing certain low doping contents. In our MnBi-5 sample, except for the highest amount of the MnBi nano-phase, a low content of pinning centers in the MnBi hard magnetic phase by interfacial diffusion of atoms from the soft magnetic phase might represent an additional reason for the as-determined increased coercive field. To investigate this idea, useful information about the interatomic diffusion is obtained for sample MnBi-5 from ^57^Fe Mӧssbauer spectroscopy, if analyzed versus the pristine FeSiB raw additive. The Mössbauer spectra collected on Fe_70_Si_10_B_20_ ribbons and the MnBi-5 sample at 5 K and 300 K respectively, are shown in Fig. 10. It can be seen that the Mössbauer spectra of Fe_70_Si_10_B_20_ ribbons at 5 K and 300 K (Fig. 10a, b) consist of broad sextets (BS) specific to the amorphous phase, which were fitted by a distribution of hyperfine magnetic fields (*B*_hf_), presented in the right side of each spectrum. On the other hand, in addition to the broad sextet belonging to the amorphous FeSiB phase, two more split crystalline sextets (CS) should be considered for fitting the Mössbauer spectrum of the MnBi-5 composite sample at 5 K. By increasing the temperature at 300 K, the two CS observed at low temperature collapse into a central doublet (CD) which superpose over the BS specific to the soft FeSiB phase. Hence, the additional sextets from the low temperature spectrum of MnBi-5 sample, have to be assigned to very fine Fe based clusters, which became superparamagnetic at 300 K, giving rise to the CD pattern. The hyperfine parameters and the relative phase component, as obtained from fitting the spectra are presented in Table 4. According to the values of the hyperfine parameters and especially to the value of the hyperfine magnetic fields, the two crystalline sextets of low temperature collapsing in the central doublet in the 300 K spectrum of sample MnBi-5 have to be assigned to Fe oxide clusters. Most probably these are formed at the grain boundaries of the MnBi nanograins, uncoupled to the soft magnetic phase. Some 18 at. % of Fe from the soft magnetic phase participate in formation of Fe oxide clusters which are enough small to be observed by XRD. Accordingly, they represent roughly 1 wt.% from the sample, which is not negligible if compared to the 25 wt. % of the MnBi nanophase in sample MnBi-5. Such Fe oxide clusters may represent on one hand the reason for the lack of exchange spring-like coupling between the MnBi nanophase and the soft magnetic phase, and on the other hand, the reason for an additional increase of the coercive field of the uncoupled MnBi nano-phase by pinning centers. A last observation concerns the typical AFM coupling between the Fe ions specific to such ultra-fine Fe oxide clusters. As a consequence, some 18% lower magnetization is expected for the soft magnetic phase which is depleted by the Fe atoms which form the AFM Fe oxide clusters. Hence, a correspondingly lower saturation magnetization of sample MnBi-5 can be inferred, again in agreement with Fig. 7b.

**Table 4:** Hyperfine parameters and relative spectral area from fitting the Mössbauer spectra in Fig. 8.

Sample	Temperature (K)	Subspectra	Relative area (%)	Hyperfine field, B_hf_ (T)	Average value of B_hf_ (T)	Isomer shift, IS (mm/s)	Quadrupole splitting, QS (mm/s)
Fe_70_Si_10_B_20_	5	BS	100	30.0(2)	28.8(1)	0.14(1)	0.003(2)
	300	BS	100	26.0(2)	26.4(1)	-0.08(2)	0.009(3)
MnBi-5	5	BS	81.5(5)	31.0(2)	28.2(2)	0.20(2)	0.01(1)
		CS1	7.1(6)	51.9(2)	–	0.34(3)	-0.12(6)
		CS2	11.4(5)	46.9(2)	–	0.44(4)	-0.19(9)
	300	BS	81.9(5)	25.0(2)	26.3(2)	0.16(5)	0.01(2)
		CD	18.1(5)	–	–	0.37(5)	0.75(6)

## Conclusion

Nanocomposite bulk samples of MnBi mixed with different amounts of FeSiB (Mn_55_Bi_45_ + *x* wt. % of Fe_70_Si_10_B_20_, *x* = 0, 3, 5, 10, 20) were prepared by Spark Plasma Sintering. The effect of FeSiB on the microstructural and magnetic properties of MnBi/FeSiB nanocomposites was investigated in detail.

The SPSed bulk pristine MnBi permanent magnet (MnBi-0) has a decreased energy product as compared to the starting MnBi raw powder. Their comparative study allows to associate a higher saturation magnetization to a lower Mn/Bi ratio in the grain and a higher coercivity to less structured MnBi nanophases.

Exchange spring effects were observed in all our MnBi–FeSiB nanocomposite samples, but their extent and specifics depend in a complex manner on the composite composition and on both the morphology and composition of the MnBi coupling grains. Important are also the oxygen atoms at the grain boundaries forming antiferromagnetic Fe oxide clusters which can destroy the interfacial exchange spring couplings between the hard and soft magnetic phases. In the context of these contradictory effects, the higher coercive fields are promoted by the less crystallized MnBi nanophases, whereas stronger exchange spring effects involve better crystallized MnBi grains with adequate Mn/Bi composition. These findings promote new possibilities for designing and tuning the magnetic properties of soft-hard magnetic nanocomposites.

The present study indicates that fabrication of MnBi based nanocomposites by powder metallurgy routes (namely in this work by spark plasma sintering of powder mixtures) is of practical interest. The exploration of exchange-spring nanocomposites instead of substituted MnBi is a promising approach towards fabrication of high performance RE-free permanent magnets. For further progress, studies should pay attention to processing conditions and routes for the fine controlled fabrication of such nanomaterials that take advantage of exchange spring phenomena through an optimal composition, morphology, size and crystallinity of the composite grains as well to the specific grain boundaries.

### Supplementary Information

Supplementary Figure 1.

Supplementary Figure 2.

## Data Availability

The datasets used and/or analysed during the current study are available from the corresponding author on reasonable request.
