# Supplementary material for: Microstructure and coupling mechanisms in MnBi–FeSiB nanocomposites obtained by spark plasma sintering
Source: Sci Rep. 2024 Jul 24;14:17029. doi: 10.1038/s41598-024-67353-7 (PMC11266415; doi:10.1038/s41598-024-67353-7)
Supplement: Supplementary file 1 — Supplementary Figure 1. [file 41598_2024_67353_MOESM1_ESM.docx]

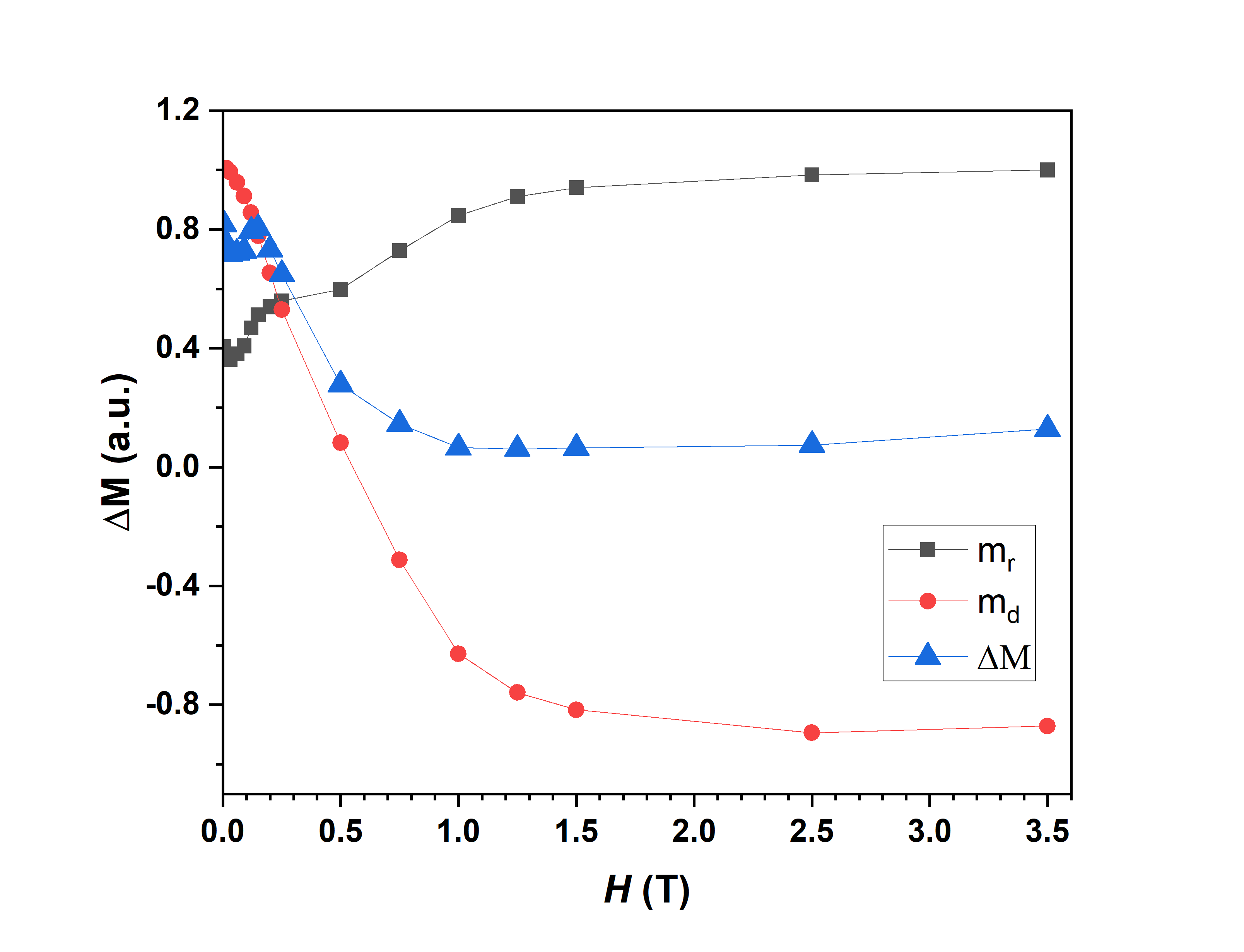


Fig.1 SM. Plot of ΔM as a function of H for sample MnBi-3, indicating the presence of a positive exchange coupling between soft and hard magnetic phases
