# Supplementary material for: Microstructure and coupling mechanisms in MnBi–FeSiB nanocomposites obtained by spark plasma sintering
Source: Sci Rep. 2024 Jul 24;14:17029. doi: 10.1038/s41598-024-67353-7 (PMC11266415; doi:10.1038/s41598-024-67353-7)
Supplement: Supplementary file 2 — Supplementary Figure 2. [file 41598_2024_67353_MOESM2_ESM.docx]

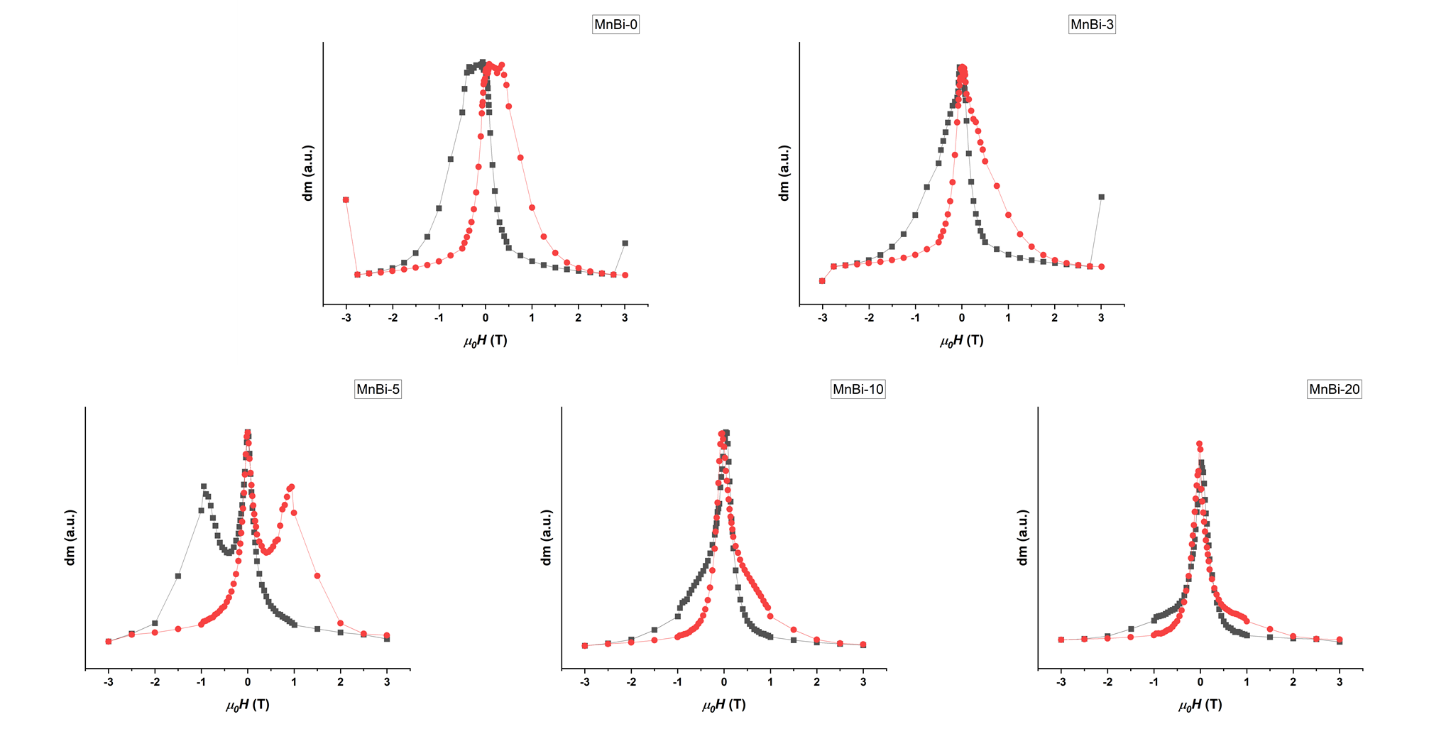


Fig. 2 SM. Plot of the first derivative of the magnetization versus the applied magnetic field for the MnBi-0, MnBi-3, MnBi-5, MnBi-10 and MnBi-20 samples used to illustrate the unique behavior with two switching fields for the MnBi-5 sample.
